# Opportunities to Impact Disability: Trends, Costs, and Evidence-Based Interventions

**DOI:** 10.1093/geroni/igab046.448

**Published:** 2021-12-17

**Authors:** Sarah Szanton, David Grabowski

## Abstract

As healthcare shifts to a focus on social determinants and population health, and older adults increasingly seek to “age in community,” it is vital to understand the functional capabilities and related costs for older adults with disability. This symposium will present data on five major areas related to older adult disability. The 1st presenter will describe recent national disability trends. The 2nd will present Medicare costs by disability, dementia, and community-dwelling status in order to illustrate how these different demographic groups vary in Medicare expenditures over time. This information is critical to policymakers and health systems leaders to plan for these populations. They will then describe a 3rd project, which employs a novel longitudinal modeling approach, Group Based Trajectory Modeling, to identify and describe the distinct trajectories of Emergency Department use after incident disability. This work assesses the heterogeneity in health care use after disability, which may be shaped by available supports. The 4th presentation will describe a combined analysis of the 11 sites that have published data from implementations of the CAPABLE program. This program is a 10 session, home-based interprofessional program that provides an occupational therapist, a nurse, and a handyworker to addresses older adults’ self-identified functional goals by enhancing individual capacity and the home environment. Taken together, these presentations can inform interventions and policies that improve the health and quality of life of older adults with disabilities.

